# Commissioning of and preliminary experience with a new fully integrated computed tomography linac

**DOI:** 10.1002/acm2.13313

**Published:** 2021-06-20

**Authors:** Lei Yu, Jun Zhao, Zhen Zhang, Jiazhou Wang, Weigang Hu

**Affiliations:** ^1^ Department of Radiation Oncology Fudan University Shanghai Cancer Center Shanghai China; ^2^ Department of Oncology Shanghai Medical College Fudan University Shanghai China

**Keywords:** beam commissioning, CT‐based IGRT, fully integrated CT‐linac

## Abstract

**Purpose:**

A new medical linear accelerator (linac) platform integrated with helical computed tomography (CT), the uRT‐linac 506c, was introduced into clinical application in 2019 by United Imaging Healthcare (UIH) Co., Ltd. (Shanghai, China). It combines a Carm linac with a diagnostic‐quality 16‐slice CT imager, providing seamless workflow from simulation to treatment. The aim of this report is to assess the technical characteristics, commissioning results and preliminary experiences stemming from clinical usage.

**Methods:**

The mechanical and imaging test procedures, commissioning data collection and TPS validation were summarized. CTIGRT accuracy was investigated with different loads and couch extensions. A series of end‐to‐end cases for different treatment sites and delivery techniques were tested preclinically to estimate the overall accuracy for the entire treatment scheme. The results of patient‐specific QA and machine stability during a one‐year operation are also reported.

**Results:**

Gantry/couch/collimator isocentricity was measured as 0.63 mm in radius. The TPS models were in agreement with the beam commissioning data within a deviation of 2%. An overall submillimeter accuracy was demonstrated for the CT‐IGRT process under all conditions. The absolute point dose difference for all the preclinical end‐to‐end tests was within 3%, and the gamma passing rate of the 2D dose distribution measured by EBT3 film was better than 90% (3% DD, 3 mm DTA and 10% threshold). Pretreatment QA of clinical cases resulted with better than 3% point dose difference and more than 99% gamma passing rate (3% DD/2 mm DTA/10% threshold) tested with Delta4. The output of the linac was mostly within 1% of variation in a one‐year operation.

**Conclusion:**

The commissioning results and clinical QA results show that the uRT‐linac 506c platform exhibits good and stable performance in mechanical and dosimetric accuracy. The integrated CT system provides an efficient workflow for image guidance with submillimeter localization precision, and will be a good starting point to proceed advanced adaptive radiotherapy.

## 1 INTRODUCTION

Since the widespread adoption of advanced radiotherapy (RT) technologies like intensity‐modulated RT (IMRT) in the 1990s,^1^ accurate and highly conformal dose delivery as well as efficient clinical workflow have become increasingly essential to medical linear accelerators (linacs). In this regard, the image‐guidance technique is indispensable for managing the geometric variations in patient set‐up and internal organ position.^2^, ^3^, ^4^ Various image‐guided RT (IGRT) systems have been employed on modern linacs, such as kilovolt (kV) and megavolt (MV) cone‐beam computed tomography (CT) (CBCT),^5^, ^6^ ^]^ fan‐beam MVCT,^7^ CT‐on‐rails,^8^, ^9^ and magnetic resonance imaging (MRI).^10^ As image guidance has become a clinical routine and the need for adaptive RT (ART) has grown, better image quality with sufficient soft‐tissue contrast is in greater demand than ever before. While MRI is the best soft‐tissue imaging modality, MRI‐guided RT systems may not be able to replace conventional X‐ray imaging systems due to technical challenges^11^, ^12^ and high startup costs.

**Fig. 1:**
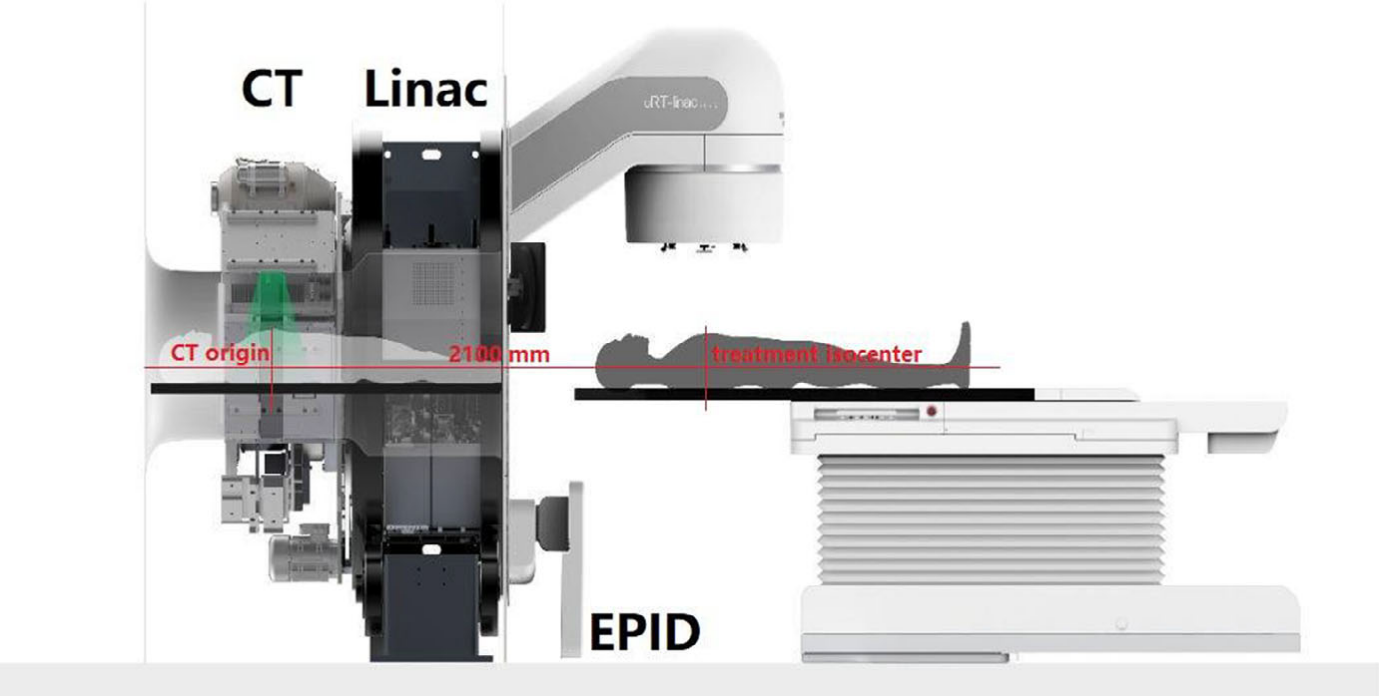
Schematic view of the uRT‐linac 506c platform.

Recently, a newly designed CT‐integrated linac named uRT‐linac 506c was introduced into the market by United Imaging Healthcare (UIH) Co., Ltd. Different from the design of a sliding CT gantry and two rotation axes of couch top in CT‐on‐rails systems,^8^ it has a diagnostic‐quality helical CT system compactly fixed behind the gantry of a C‐arm linac, and the patient is sent through the scanner by moving the couch longitudinally, as shown in Figure 1. The combination of CT and a linac enables a seamless workflow from simulation to treatment on one unit. Compared with kV‐CBCT that is commonly used for IGRT, the helical kV fan‐beam CT (FBCT) is nearly free from degrading of photon scattering that results in shading artifacts and deterioration of overall uniformity, providing a slice‐to‐slice comparison of patient's anatomy with planning CT and a direct access to online adaptive replanning with accurate CT number accounting for interfractional anatomic changes.

In 2019, the uRT‐linac 506c unit was installed and prepared for clinical operation. The objectives of this work are to (a) introduce the main technical specifications of this new treatment platform, including the mechanical precision, CT‐IGRT accuracy, and clinical workflow; (b) summarize the procedures of beam data collection and commissioning tests; (c) present the results of beam data measurements and end‐to‐end tests for the entire clinical scheme; and (d) report the quality assurance (QA) results and preliminary experience in a 1‐year operation. The present report is based on recommendations of several practice protocols and guidelines for commissioning and testing prior to clinical usage, including the American Association of Physicists in Medicine (AAPM) Task Groups (TG) reports about code of practice (No. 45^13^), multileaf collimators (MLCs) (No. 50^14^), dosimetry calibration (No. 51^15^), QA for treatment planning (No. 53^16^), film dosimetry (No. 69^17^), commissioning equipment and procedures (No. 106^18^), IMRT commissioning (No. 119^19^), QA for linac (No. 142^20^), commissioning for Monte Carlo (MC)‐based treatment planning (No. 157^21^), QA for CT‐based IGRT systems (No. 179^22^), commissioning and QA for treatment planning (MPPG 5.a^23^), tolerances and methodologies for IMRT QA (No. 218^24^), and IAEA Technical Reports Series (TRS) No. 430^25^ about commissioning and QA of computerized planning systems, as well as publications on commissioning procedures of linacs from single or multiinstitutional investigations.^26^, ^27^, ^28^, ^29^, ^30^

## 2 BACKGROUND

**Fig. 2:**
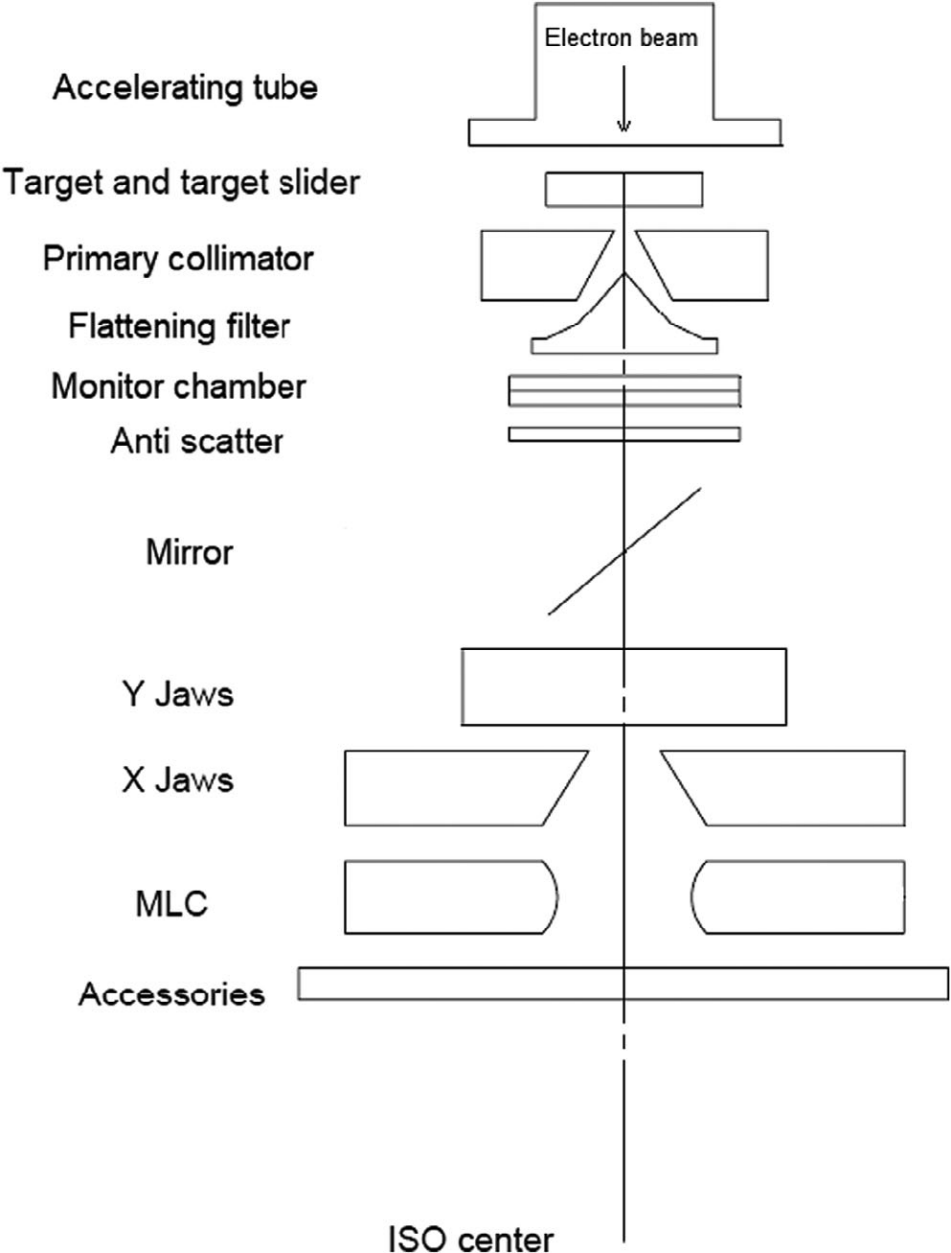
Diagram of the inner beam‐line components of the uRT‐linac 506c treatment head.

Figure 1 shows a schematic view of the uRT‐linac 506c platform, and the main features are summarized in Table 1. It has a 16‐slice helical CT imager coaxially attached to the gantry of a C‐arm linac, with a designated longitudinal distance of 2100 mm between the treatment isocenter and CT origin. The enclosed CT bore has a diameter of 70 cm. The gantry of the linac is capable of one and a half revolutions, namely, from −362° to 182°, which is achieved by using a long slack in the cables wrapping around the barrel of the gantry. Diagram of the beam line inside the treatment head is shown in Figure 2. The accelerating tube is mounted with its axis parallel to the central axis of the radiation beam such that electron beam is accelerated and strikes the target without bending. The linac is designed to generate and deliver photon beams of two energies, i.e., the 6‐MV treatment beam and the 1.5‐MV imaging beam, which are produced by electron beams with peak energy of 6‐ or 1.5‐MeV incident on a high‐Z or low‐Z target, respectively. Since there is no beam steering, focusing nor bending, it is assumed that the electron beams of two energies hit the same position of the targets, in other words, the imaging beam and treatment beam are generated equivalently by the same source. The 6‐MV treatment beam is delivered in flattened (with flattening filter, FF) and unflattened (flattening‐filter‐free, FFF) modes with a maximum dose rate of 600 and 1400 MU/min, respectively, while the 1.5‐MV imaging beam is in FFF mode with a dose rate of 40 MU/min. Clinical electron beam modality is not available on this machine. The treatment head is equipped with dual‐layered collimating jaws and 60 pairs of MLCs with a 0.5‐cm width at the isocenter in the inner 20 cm and a 1.0‐cm width in the outer 20 cm, projecting a maximum field size of 40 × 40 cm^2^. The available delivery techniques include three‐dimensional conformal radiation therapy (3D‐CRT), step‐and‐shoot IMRT (sIMRT), dynamic IMRT (dIMRT), and volumetric modulated arc therapy (VMAT, named uARC on this platform with “u” the initial of UIH). Virtual wedges of 10°/15°/20°/25°/30°/45°/60° are provided using dynamic jaws. The image acquisition system consists of MV portal and MV CBCT by employing the 1.5‐MV imaging beam and an amorphous silicon electronic portal imaging device (aSi‐EPID, 40 × 40 cm^2^ active area) and kV FBCT offered by the integrated CT system. A treatment couch with movable base is utilized for patient transportation and IGRT automatic correction. It is a four‐degrees‐of‐freedom (4DOF) couch allowing only translations (lateral, longitudinal, vertical) and yaw rotation. To mitigate risks of collision, there are triple protection modules in software and hardware: one is a trajectory monitoring module used to avoid collisions between mechanical components of the machine (patient is not taken into account in this module), another is an infrared laser proximity sensor mounted on the gantry to assure enough clearance for patients, and the third is a ring of spring‐loaded mechanical switches fixed on the EPID holder that supports the panel to protect the detector from being touched by the casing. An interlocking signal will be triggered immediately to stop motion in case that any of the modules predicts/detects a collision. The gantry isocenter clearance is 46.7 cm in radius at most and the patient clearance is limited to 43 cm by the proximity sensor.

**Table 1:** Description of the uRT‐linac 506c platform.

Feature	Description
Configuration	C‐arm linac integrated with 16‐slice helical CT
Enclosed CT bore	70 cm in diameter
Linac gantry rotation	−362° to 182°, 7°/s rotation speed at maximum
Treatment beam	6 MV (FF/FFF) X ray
Nominal dose rate	25–600 MU/min (FF), 75–1400 MU/min (FFF)
MLC design	1.0 cm × 20 pairs, 0.5 cm × 40 pairs, 2.5 cm/s leaf‐only speed, 5.5 cm/s with carriage together
Maximum field size	40 × 40 cm^2^
Delivery techniques	3D‐CRT, sIMRT, dIMRT, uARC
Imaging systems	MV portal, MV‐CBCT, kV‐FBCT
Treatment couch	4‐DOF carbon fiber couch with movable base

**Fig. 3:**
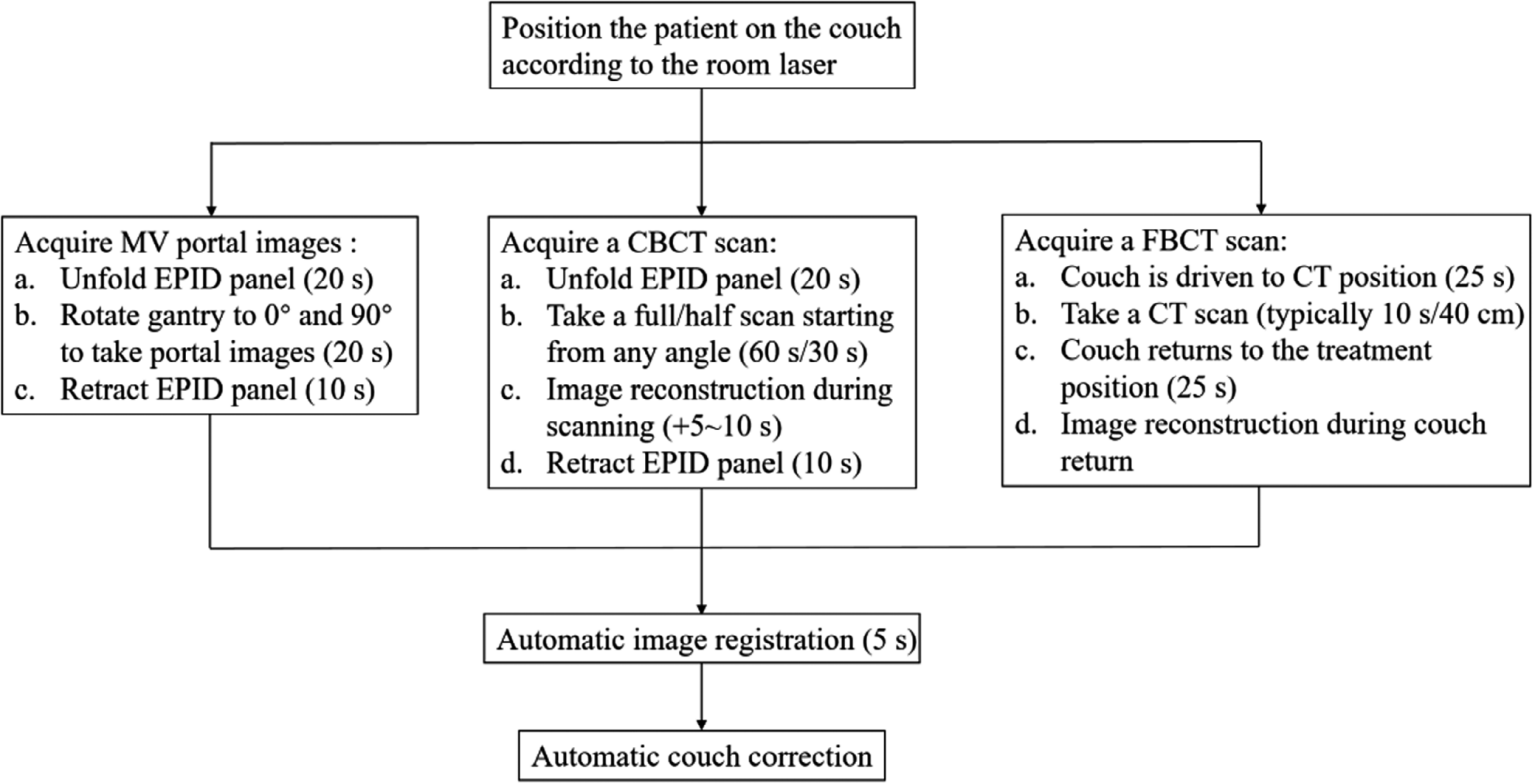
Clinical IGRT workflows of three imaging techniques on the uRT‐linac 506c.

**Fig. 4:**
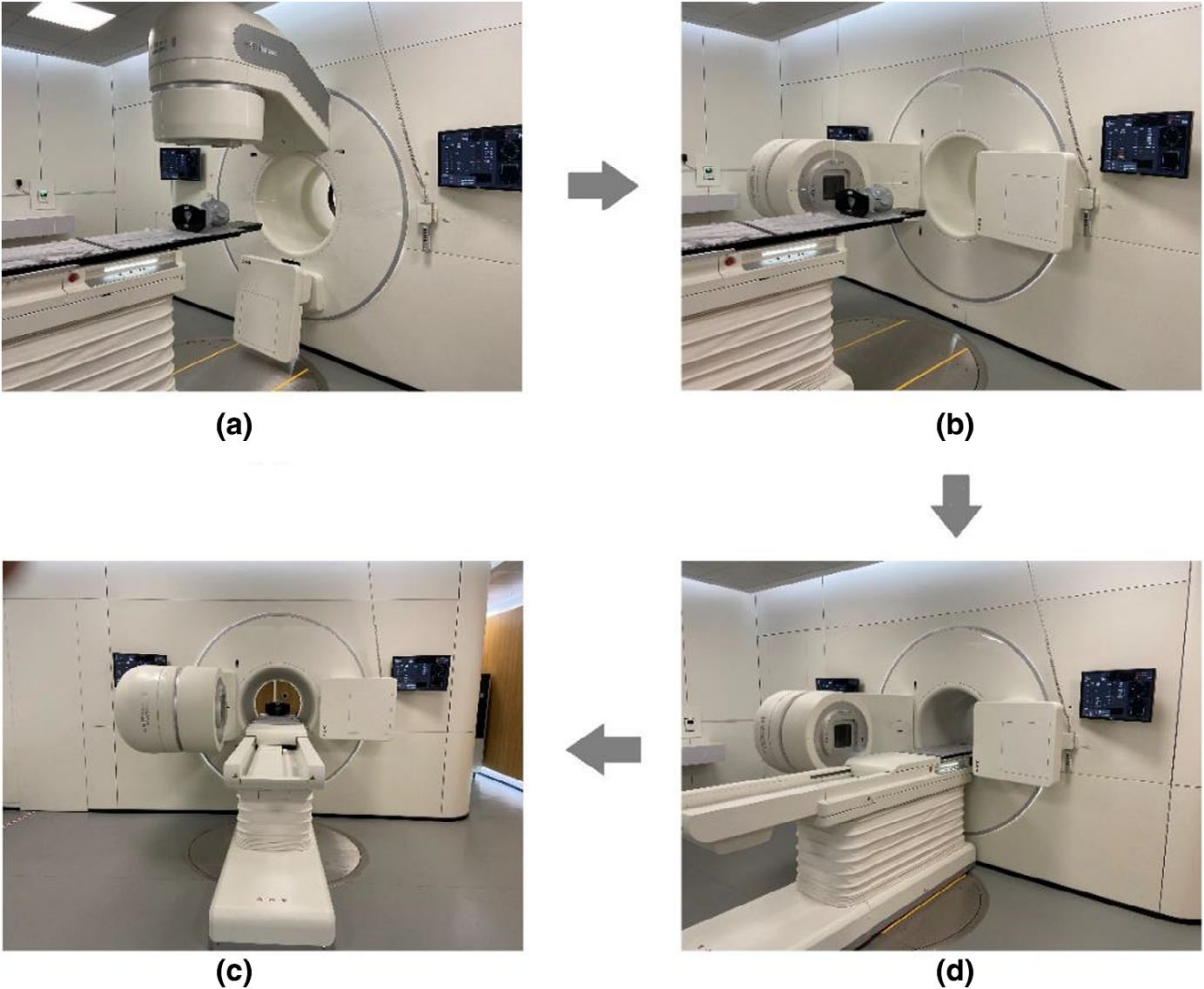
Room‐view photos of FBCT acquisition. (a) Patient is positioned at the treatment position. (b) Gantry is rotated to 270°, and meanwhile, (c) couch base is driven close to the gantry and couch top is translated to the CT bore. (d) FBCT scanning is started.

The integrated CT scanner is available as a conventional simulator when required. In other cases, it is used for image guidance prior to treatment. Figure 3 illustrates the clinical IGRT workflow of the three imaging techniques. The amount of time taken for each step is indicated in brackets. MV portal/CBCT procedures are similar to those on other linacs. In regard to the FBCT acquisition, one needs to press a button of “Go to CT position” to send the patient into the CT bore first, by rotating the linac gantry to 270°, meanwhile driving the couch base close to the gantry and translating the couch top to the CT position, as shown by the room‐view photos in Figure 4. For purpose of IGRT, scout scan is not needed due to the presence of planning CT, and scanning protocol is specified only once for each patient. The subsequent procedures are accomplished with nearly one click, which is greatly simplified compared with conventional CT scanning. Typical scanning time for a range of 40 cm is around 10 s (19.2‐mm collimator width, 0.5 s/rotation, 120 kV, 200 mAs). Images are reconstructed in parallel with couch returning to the treatment position by pressing a button of “Out of CT position.” 4D FBCT can be acquired by using a bellyband‐type sensor, and belt‐regulated respiratory gating treatment delivery for motion management is also supported. 4D‐FBCT IGRT may take longer, from 5 to 10 min depending on protocols. The acquired planar/volumetric images are registered to the planning CT using 2D/3D registration algorithms based on either the bony anatomy or the soft tissues (with manual adjustment if necessary). 4D‐FBCT IGRT is performed by average‐to‐average comparison. The registration algorithms provide couch corrections in three translational and three rotational (optional for 3D) directions. The treatment couch is then driven to the target position automatically. Although only translational corrections are allowed, the rotational information could serve as a reference for patient repositioning.

For the running of uRT‐linac 506c, existing commercial treatment planning systems (TPSs) or oncology information systems (OISs) are currently not supported for use. The linac has its own control system, consisting of an integrated TPS + OIS platform named uRT‐TPOIS and a treatment delivery system (TDS). The former manages the clinical workflow including patient registration, contouring, plan creation and evaluation, patient QA, plan scheduling, and image review, and the latter is used for interactions with the linac and the CT scanner, as well as for radiation delivery.

## 3 METHODS AND METERIALS

### 3.A Isocenter verification

A conventional spoke‐shot test was performed to investigate the coincidence between the mechanical isocenter and the treatment isocenter during gantry and collimator rotations. The mechanical isocenter was indicated using the front pointer and marked by a pinhole on the film, while the intersection of the central axes of the beams (or the point with the shortest distance to all beam axes) defined the treatment isocenter. As required in TG‐142,^20^ deviation between the two isocenters should be less than 1 mm. For couch rotation, a radiopaque ball bearing (BB) was placed on the couch near the isocenter, and portal images of a 10 × 10‐cm^2^ field were acquired by EPID during couch rotation. The distance between the center of the BB trajectory and the center of the radiation field was required to be no more than 0.25 mm, which is in compliance with the manufacturer's specifications.

We further verified the isocentric coincidence by an independent EPID‐based Winston–Lutz (WL) test at various gantry and couch angles. The WL phantom (BB of 5 mm in diameter) was set to the mechanical isocenter by aligning to the crosshair at gantry 0° and 90°, and then moved to the calculated isocenter location by using images at four cardinal gantry angles with opposing collimator angles.^28^, ^30^ After aligning the BB location to the radiation isocenter precisely, we sampled six oblique gantry angles (30°, 60°, 130°, 230°, 300°, and 330°) and three couch angles (0°, 90°, and 270°). On each EPID image, the crossline and inline deviations (ΔU and ΔV) between the center of the jaw‐defined 2 × 2‐cm^2^ aperture and the center of the BB were determined. The confinement radius of central beam axis variation during gantry and couch rotations was defined as the maximum 2D centroid distance ($\Delta R=\sqrt{{\left(\Delta U\right)}^{2}+{\left(\Delta V\right)}^{2}}$, rescaled to the isocenter plane).

### 3.B Commissioning data collection

The required data for beam commissioning included percent‐depth dose (PDD), lateral profiles, output factors, MLC transmission factor, and leaf‐tip offset for 6XFF and 6XFFF modes. An IBA Blue Phantom 2 water tank controlled by OmniPro‐Accept 8 software (IBA Dosimetry GmbH, Germany) was used for beam scanning and data collection, following recommendations from TG‐106.^18^ The PDD, crossline/inline profiles and output factors were measured at a source‐to‐surface distance (SSD) of 100 cm for field sizes ranging from 1 × 1 cm^2^ to 40 × 40 cm^2^. Beam profiles by jaw‐only collimation and by jaw‐and‐MLC collimation were both measured at depths of *d*_max_, 5, 10, 20, and 30 cm. Output factors were acquired with jaw‐only collimation at a depth of 5 cm for all field sizes. The MLC transmission factor, defined as ratio of dose with block MLC field to open field, was measured for both leaf banks at 100‐cm SSD and a depth of 1.5 cm. The MLC leaf‐tip offset is one of the parameters for MLC modeling in uRT‐TPOIS, which is referred to as the offset of the nominal leaf position readout from the actual position defined by the radiation field. Details about the MLC modeling will be discussed in the later subsection. The leaf‐tip offset was measured as a function of leaf position, by acquiring the crossline profile of an 8 × 20‐cm^2^ MLC‐defined field at 90‐cm SSD and 10‐cm depth, sweeping over the entire range of leaf travel with a step size of 4 cm. The edge of actual radiation field was determined by the 50% intensity point relative to the central value of the profile for flattened beam and by the inflection point of the penumbra region for unflattened beam, respectively.

Two types of detectors were used during commissioning measurements. For a field size of ≤5 × 5 cm^2^, a single‐crystal diamond detector (microDiamond 60017, 0.004 cc, PTW, Freiburg, Germany) was chosen because of its high spatial resolution and low volume averaging effect. The beam data for field sizes of ≥5 × 5 cm^2^ were collected using medium‐sized cylindrical ion chambers (Scanditronix CC13, 0.13 cc, IBA). For a field size of 5 × 5 cm^2^, both ion chamber and diamond detector measurements were performed for cross‐validation and output factor renormalization. The MLC transmission factor and leaf‐tip offset were measured using the diamond detector. In addition, a 0.6‐cc Farmer chamber (PTW 30013) was used for absolute dose calibration at *d*_max_.

### 3.C Beam modeling and IMRT commissioning

The measured beam data were imported into the uRT‐TPOIS for beam modeling. The uRT‐TPOIS provided dose calculations based on collapse cone convolution (CC) and MC methods and was commissioned following the recommendations of TG‐53,^16^ TG‐157,^21^ and MPPG 5.a.^23^ Here, we focus on validation of the beam models, without going into too much detail about nondosimetric commissioning. In basic beam modeling, the TPS model parameters were iteratively adjusted to optimally agree with the measured data in the high‐dose region, penumbra, and low‐dose tail regions, within specific tolerance values and evaluation criteria.^21^, ^23^ Note that the statistical uncertainty in the MC calculation was user‐defined and it was set to 1% throughout this article unless otherwise specified.

**Fig. 5:**
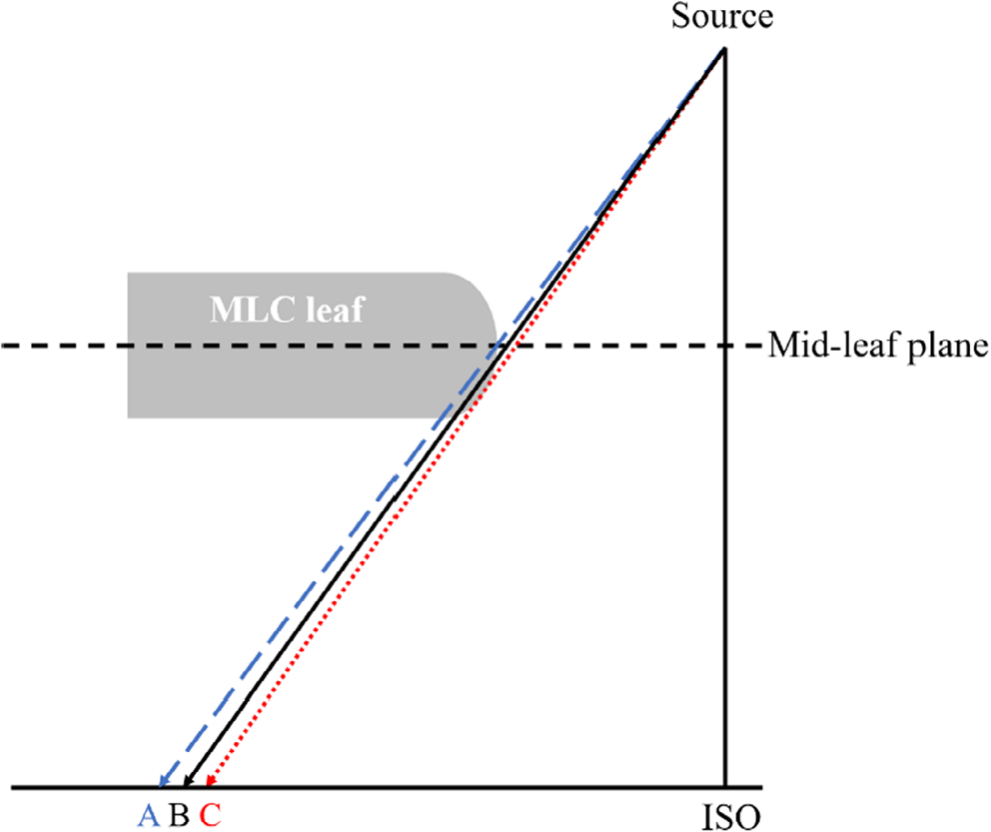
Position of rounded‐end MLC leaf at the isocenter plane in different definitions, i.e., projected leaf position A (defined by the center of leaf tip), physical leaf position B (defined by the radiation field) and geometric leaf position C (defined by the light field).

Special considerations have been given for modeling the characteristics of curved‐end MLC, as it is known that small changes in leaf‐tip position may lead to large dose deviations in IMRT plans.^31^, ^32^ As proposed by Vial et al.,^33^ the rounded end of MLC leaf tip shows positions at the isocenter plane in different definitions, i.e., projected leaf position A (defined by the center of leaf tip and used to control leaf motion), physical leaf position B (defined by the radiation field), and geometric leaf position C (defined by the light field), as illustrated in Figure 5. In the MLC control system of the uRT‐linac 506c, the leaf position was calibrated so that the physical leaf position corresponded to the nominal leaf position, namely, the radiation field size was consistent with the digital field setting, by using a vendor‐supplied calibration table. The light field was adjusted to coincide with the radiation field by shifting the light source. In order to take into account the limitations of the standard calibration procedure, the MLC leaf‐tip offset was determined by the radiation field measurements mentioned above, and the leaf position used for planning was shifted by the value of the offset so that the calculated treatment field in uRT‐TPOIS matched the actual radiation field. Furthermore, the tongue‐and‐groove effect and the additional transmission through the rounded leaf edge were accounted for by another two parameters called tongue‐and‐groove width and leaf‐tip width, respectively, which were predefined by the vendor in the MLC model.

The TPS models were almost finalized after basic beam modeling was done, without the need of further adjustment by IMRT QA, probably owing to the thorough modeling of physical measurements based on the multiparameter MLC model. As recommended in MPPG.5.a,^23^ basic beam validation was performed for each model with field configurations that were different from those used for modeling. Specific open‐field point doses were measured in the water tank and compared with TPS calculations using the photon tests from IAEA TRS No. 430.^25^ The CC13 ion chamber was used for point dose measurements, whose reading was calibrated using the machine output at *d*_max_. The TPS models were further validated using a series of test cases that are representative of typical clinical treatments from simple to complex modulations provided in TG‐119 test suite.^19^ A PTW slab phantom in water‐equivalent RW3 material with dimensions of 30 cm × 30 cm × 30 cm was used for dose measurements. The absolute point dose differences (DDs) were measured in both the target (PTV) region and organ at risk (OAR) region using a 0.125‐cc ion chamber (PTW 31010 Semiflex) placed at a depth of 15 cm. Planar dose measurements were conducted using Grafchromic EBT3 films (Ashland Advanced Materials, Bridgewater, NJ) and ArcCHECK (Sun Nuclear Corporation, Melbourne, FL).

### 3.D Imaging tests

Technical specifications of the imaging devices on the uRT‐linac 506c unit are described in Table 2. Here, the imaging dose of the MV‐CBCT or kV‐FBCT was estimated by assessing the volume CT dose index (CTDI_vol_) for each protocol, and the MV portal dose was roughly calculated from the MV‐CBCT dose per monitor unit. For the purpose of image guidance rather than diagnosis, low‐dose FBCT protocols are available to minimize additional radiation dose to patients. Currently, the imaging dose is not accounted for in treatment planning. During commissioning, the image quality and geometric accuracy, namely, imaging/treatment coordinate coincidence, of the devices were investigated based on the QA protocols in TG‐179.^22^

**Table 2:** Technical specifications of imaging devices on uRT‐linac 506c.

Imaging beam		MV portal	MV‐CBCT	kV‐FBCT
		1.5‐MV FFF	1.5‐MV FFF	70–140 kV
Detector	Type	aSi‐EPID	aSi‐EPID	GOS+Si CT detector
	Dimension in pixels	1024 × 1024	1024 × 1024	816 × 24
	Pixel size	0.4 mm ≈0.28 mm @ISO	0.4 mm ≈0.28 mm @ISO	1.0 mm;; ≈0.6 mm @ISO
Spatial resolution		0.28 mm @ISO	0.78 mm @512 × 512	0.97 mm @512 × 512
Field of view (FOV)		27 cm	40 cm	50 cm
Scanning range		27 cm	27 cm	100 cm
Imaging dose		~1 mGy	10–30 mGy	Diagnostic: 10–50 mGy
				Low‐dose: 3.5–5 mGy

#### 3.D.1 Image quality

**Fig. 6:**
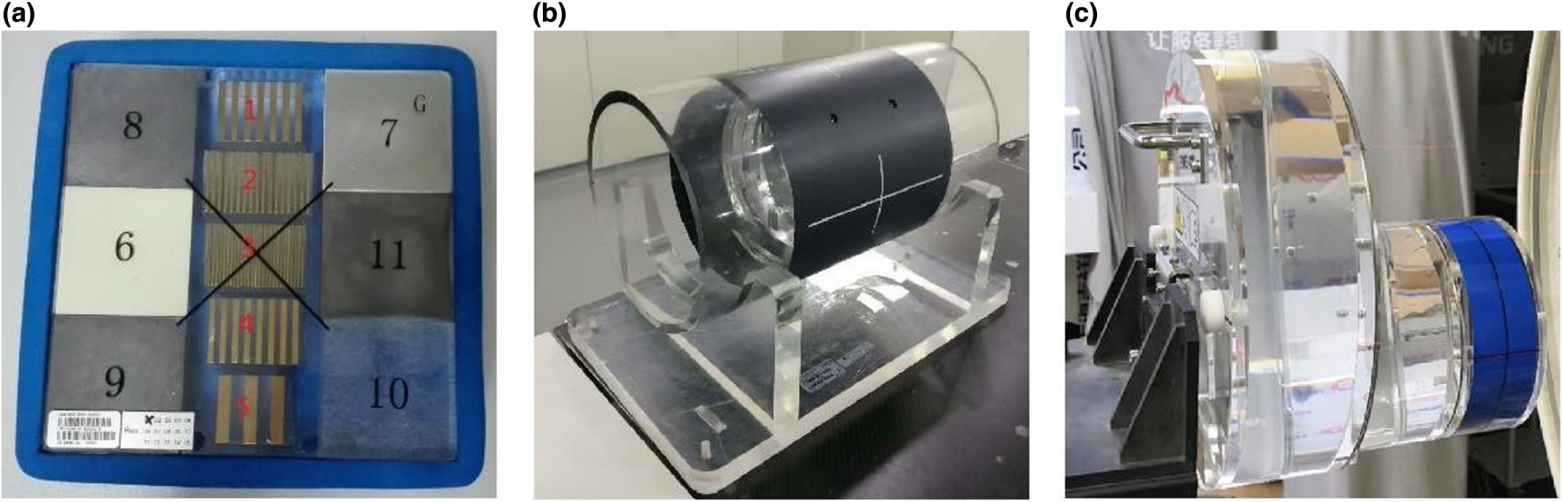
(a) 2DQA phantom for portal imaging, (b) 3DQA phantom for CBCT and (c) CT system phantom for FBCT.

The image quality of the IGRT systems was assessed by evaluating the spatial resolution and soft‐tissue contrast. Figure 6a–c shows the vendor‐provided phantoms for imaging QA, i.e. 2DQA phantom for portal imaging, 3DQA phantom for CBCT and CT system phantom for FBCT, respectively. The spatial resolution was determined by the visually resolvable line‐pair (lp) information on the acquired image, while the contrast‐to‐noise ratio (CNR) was measured by various contrast media in the phantoms. For comparison with the vendor's results, the CatPhan604 phantom (The Phantom Laboratory, NY) was also used to investigate the spatial and contrast resolution, image uniformity, and low contrast detectability of volumetric CBCT and FBCT. In addition, the CT number of FBCT was calibrated utilizing the electron density phantom (model 062 M, CIRS, Norfolk, VA, USA) for the use of dose calculation.

#### 3.D.2 Geometric accuracy

Coordinates of the imaging systems should be aligned to those of the linac to deduce the positional deviation of the IGRT image relative to the treatment isocenter. For MV portal/CBCT, the isocenter alignment of the 1.5‐MV FFF imaging beam with the linac was tested using a similar WL procedure in Section 3.A, by acquiring the EPID images with the imaging beam instead of the treatment beam. Besides, the positioning accuracy of the EPID panel during gantry rotation was also investigated. In order to align the portal image/reconstructed volumetric dataset with the treatment isocenter, the positioning error of the flat panel due to component flexing was corrected in software by shifting the projection images according to a vender‐provided flex map, which described the variation of EPID position as a function of gantry angle. At commissioning time, the positioning repeatability of EPID was evaluated using a panel alignment procedure that was implemented by inserting an imaging reticle accessory with two orthogonal tungsten wires. The projection images of the reticle were acquired at various gantry angles, and the positional accuracy of the EPID was deduced by comparing the position of the projected crossing with the position of the central pixel that was corrected to nominally intersect with the isocenter. The panel alignment test is also part of routine weekly QA to regularly check the EPID positioning reproducibility.

For kV‐FBCT, the CT scanner and the linac do not share an isocenter. The distance from the treatment isocenter to the CT origin is covered by the base moving of the couch (1300 mm) and couch extension (800 mm), as shown in Figures 1 and 4, for which the effects of couch sag and flex cannot be ignored. A real‐time correction mechanism has been built in software to correct these shifts, which is achieved using a laser ranging system mounted at the bottom of the linac gantry and an N‐shape radiopaque wire implanted in the couch board. The couch level at the treatment position is recorded by the range finder, and the N‐shape wire is used to localize the couch position in CT series. By tracking the positions of the couch with respect to the linac isocenter and CT origin respectively, the coordinates of CT series are corrected to align to those of the linac within an error of 0.5 mm, according to the manufacturer's specification.

Also, we have performed end‐to‐end tests using the Multiple Imaging Modality Isocentricity (MIMI) phantom (Standard Imaging, Middleton, WI) to investigate the overall IGRT accuracies of the imaging systems, which involved a combination of errors in laser/treatment/imaging coordinate alignment, image registration, and remote control of couch movement. Note that the room lasers were adjusted to be used as a surrogate of the treatment isocenter. The MIMI phantom was featured with two sets of cross‐hair markers, corresponding to the center of the phantom and the decentered position that was offset with known distances of 12, 10, and 14 mm in the X, Y, and Z directions, respectively. The test procedures were as follows.

1. Prior to IGRT use, the CT image of the MIMI phantom was acquired as a reference, and a 3DCRT plan was generated based on the contouring of an arbitrary target volume.
2. Loads of 40 or 100 kg were placed on the couch. The phantom was positioned with different couch extensions and aligned to the isocenter with the help of the room laser. The setup error was corrected each time by applying the couch shifts based on initial CBCT localization.
3. At each couch position, the MV portal image, MV CBCT, and kV FBCT were acquired in turn and registered to the reference CT, respectively. The results of couch corrections for the three types of images were recorded accordingly.
4. With loads of 100 kg and full couch extension, the phantom was displaced by the predefined distances with respect to the isocenter (by aligning the decentered marker to the laser), and step (3) was repeated.

### 3.E End‐to‐end verification

We performed 45 end‐to‐end tests for different treatment sites and delivery techniques to validate the dosimetric accuracy throughout the entire clinical workflow, including CT simulation, treatment planning, target localization, and plan delivery. Two anthropomorphic phantoms designed for stereotactic QA were used in this step. A head and shoulder phantom (model 136500, CIRS, Norfolk, VA, USA) was used for head and neck (H&N) cases, and a thorax phantom (model 036S, CIRS, Norfolk, VA, USA) was used for breast and lung cases. Both phantoms provided ion chamber cavities for point dose measurements and separable portions for film dosimetry. Due to unavailability of a pelvis phantom that was capable of ion chamber measurements, the head and shoulder phantom was utilized alternatively to test the rectum and cervix cases during the commissioning process. The phantom patients were CT‐scanned in 1‐mm slice thicknesses. Corresponding clinical plans were copied to the phantom images and calculated for dose distribution. Note that the inserts for film dosimetry in the thorax phantom were not located near the treatment target for breast cases and some of lung cases; therefore, the film analysis of these plans was performed using a stereotactic dose verification phantom (SDVP) with a heterogeneity insert (Standard Imaging, Middleton, WI) instead. For lung cases using stereotactic body RT (SBRT) technique, the tumor volume inside the left or right lung of the thorax phantom was delineated as the treatment target, and SBRT plans were developed using 6XFFF beams following the clinical criteria.

The phantoms were set up and localized by CBCT/FBCT. The planned doses were delivered and measured using a 0.125‐cc ion chamber (PTW 31010 Semiflex) and Grafchromic EBT3 films. The exposed film was scanned and converted into dose map following the film scanning guidelines and calibration procedures^17^ and then compared with the planned dose distribution at the level of film using FilmQA Pro 2016 software (Ashland Advanced Materials, Bridgewater, NJ).

### 3.F Routine QA

During the commissioning of the uRT‐linac 506c, a set of baseline values for mechanical and dosimetric QA were established. Prior to clinical operation, the machine output was validated by third‐party testing from a local institute. Routine QA was performed according to the recommended frequencies and tolerances in TG‐142.^20^ Additional QA tasks for the CT‐based IGRT system including imaging quality, CT number stability and geometric accuracy were implemented based on recommendations of TG‐179.^22^ More specifically, we performed monthly QA tests of imaging quality using the CatPhan604 phantom, and weekly QA tests of IGRT accuracy using the MIMI phantom, following the procedures in Section 3.D. Besides, the vendor provides built‐in modules of machine QA in the TDS, such as panel alignment, portal 2DQA, CBCT 3DQA, and CT system QA, as mentioned earlier, as well as MLC/jaw position QA and beam output QA based on EPID. The QA modules provide instant result analysis based on customized phantoms and test plans with a similar precision as given by third‐party vendors. Systematic machine performance was recorded in the QA modules during the commissioning period and used as a reference for daily/weekly QA. Trending of the stored QA data could be a help to identify any potential problem.

Patient‐specific QA was performed for each clinical plan using a 0.6‐cc Farmer chamber (PTW 30013) and Delta4 phantom (ScandiDos, Uppsala, Sweden). To minimize the effect of volume averaging, the chamber was placed in the area with reasonably uniform dose distribution, where the maximum and minimum doses across the volume were within 3% of the mean chamber dose. The tolerance limit for gamma evaluation was 95% of points passing the criteria of 3% DD and 2‐mm DTA with 10% threshold based on the recommendations of TG‐218.^24^

## 4 RESULTS

### 4.A Isocentricity

The results of the spoke‐shot tests indicate that in all gantry and collimator settings, the distance between the mechanical isocenter and the treatment isocenter was well within 1 mm, approximately 0.6 mm during gantry rotation and 0.2 mm during collimator rotation. For couch rotation, the distance was measured as 0.14 mm by the BB test.

The EPID‐based WL data were analyzed by an in‐house MATLAB tool, as shown in Table 3. The radius of isocentricity was 0.63 mm as determined by the maximum 2D distance at gantry/collimator/couch angles of G330°/C0°/T270°.

**Table 3:** WL results of the in‐house analysis (rescaled to the isocenter plane).

G: 130°	C: 0°	T: 0°	G: 230°	C: 0°	T: 0°	G: 300°	C: 0°	T: 0°	G: 60°	C: 0°	T: 0°
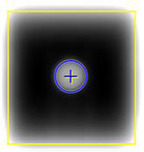			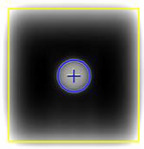			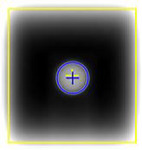			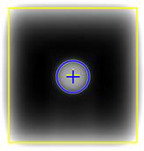		
ΔU (mm):		0.13	ΔU (mm):		‐0.06	ΔU (mm):		−0.07	ΔU (mm):		−0.01
ΔV (mm):		0.18	ΔV (mm):		‐0.10	ΔV (mm):		−0.36	ΔV (mm):		−0.21
ΔR (mm):		0.22	ΔR (mm):		0.12	ΔR (mm):		0.36	ΔR (mm):		0.21

G: 130°	C: 0°	T: 270°	G: 330°	C: 0°	T:270°	G: 230°	C: 0°	T: 90°	G: 30°	C: 0°	T: 90°
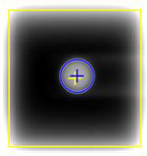			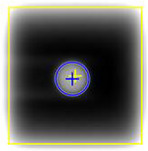			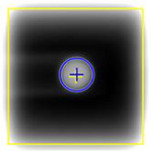			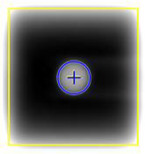		
ΔU (mm):		−0.33	ΔU (mm):		0.45	ΔU (mm):		−0.16	ΔU (mm):		−0.08
ΔV (mm):		0.33	ΔV (mm):		−0.45	ΔV (mm):		0.28	ΔV (mm):		−0.16
ΔR (mm):		0.47	ΔR (mm):		0.63	ΔR (mm):		0.33	ΔR (mm):		0.18

Note

The edge and center are shown in yellow for the radiation field and in blue for the BB. ΔU, ΔV, and ΔR denote the crossline (left−/right+), inline (superior−/inferior+) offsets, and 2D distance between the two crosses, respectively.

Abbreviations: C, collimator angle; G, gantry angle; T, couch angle.

### 4.B Commissioning data and TPS validation

#### 4.B.1 PDD and profile agreements

**Fig. 7:**
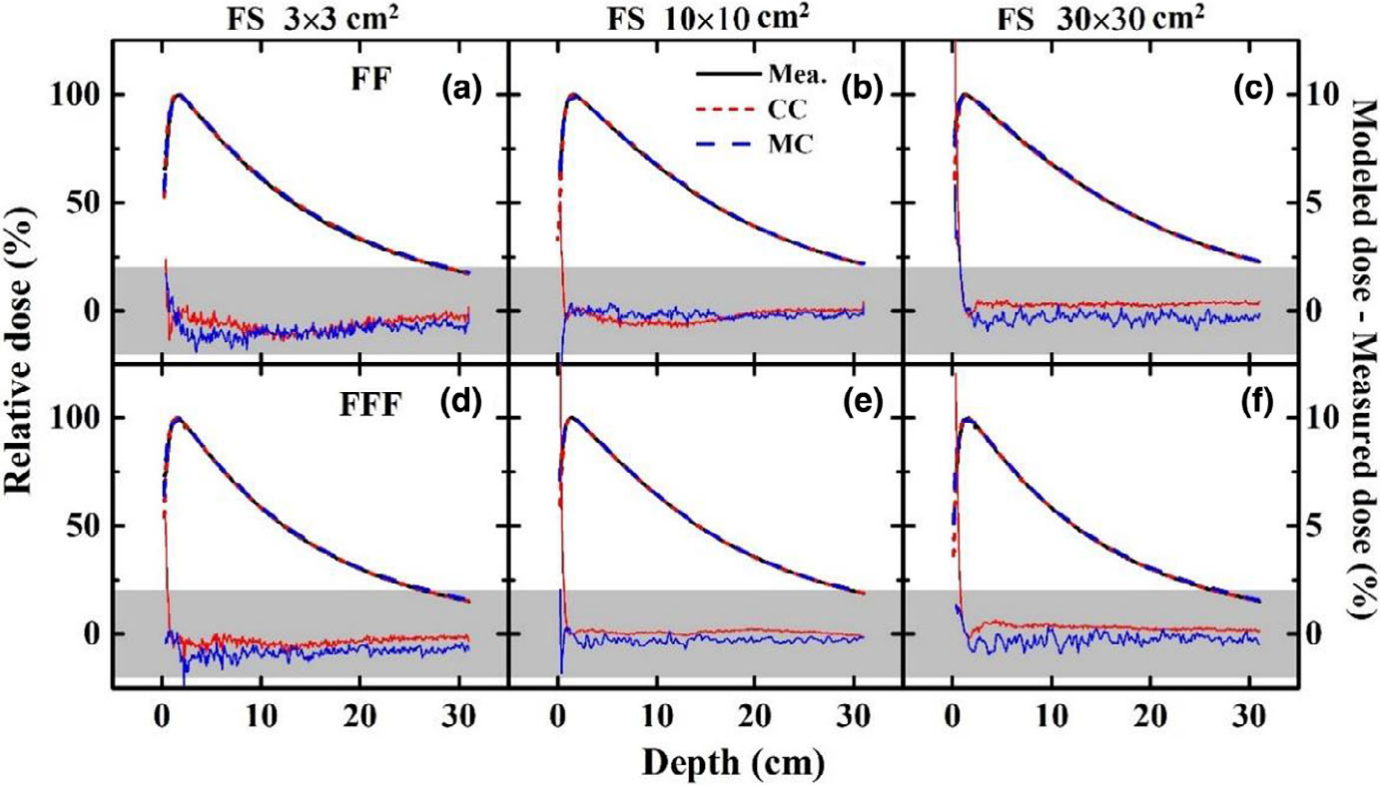
Agreements and relative differences of PDDs for 3 × 3 (left), 10 × 10 (middle), 30 × 30 (right) cm^2^ fields between TPS calculations (CC and MC) and beam measurements for 6XFF (a, b, c) and 6XFFF (d, e, f). The shaded area represents the region within a relative difference of 2% between the modeled dose and measured dose.

**Fig. 8:**
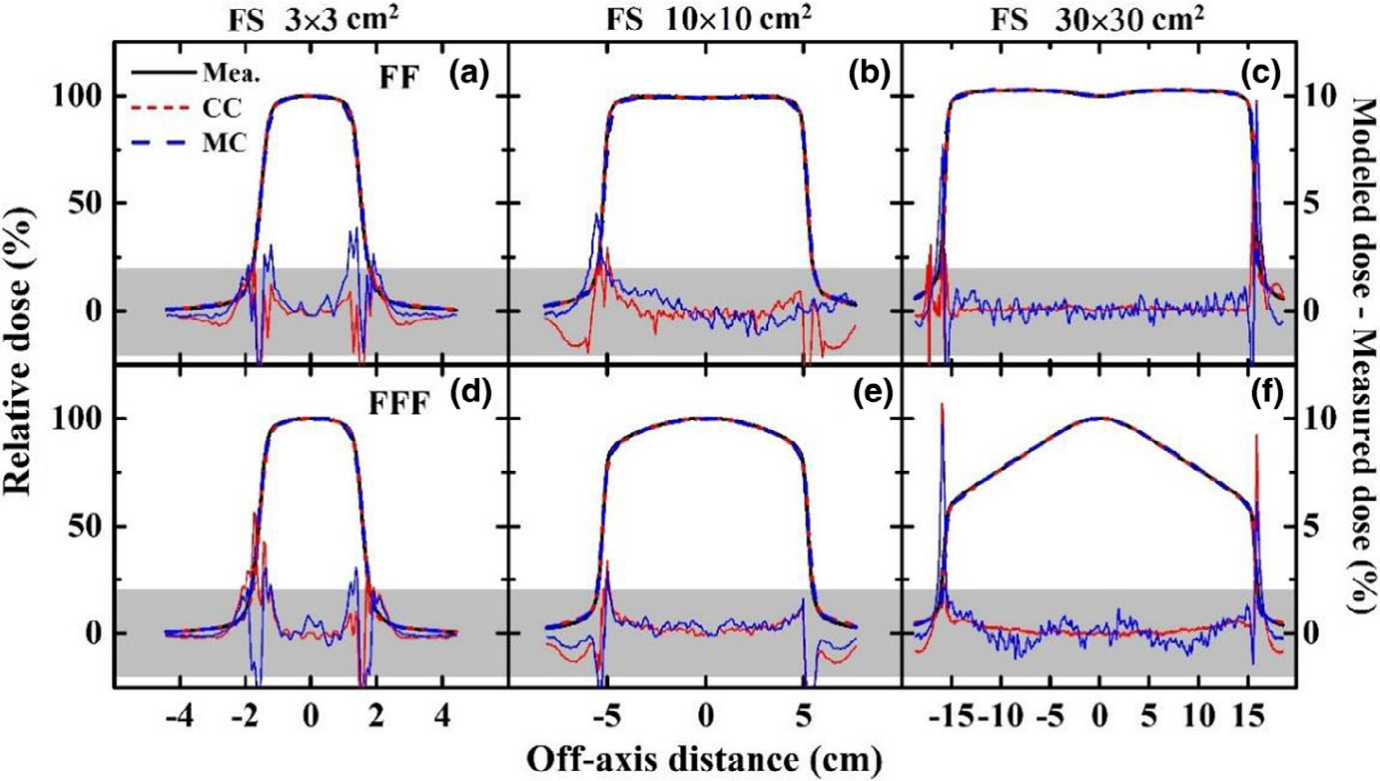
Agreements and relative differences of profiles for 3 × 3 (left), 10 × 10 (middle), 30 × 30 (right) cm^2^ fields (SSD = 100 cm, depth = 5 cm) between TPS calculations (CC and MC) and beam measurements for 6XFF (a, b, c) and 6XFFF (d, e, f). The shaded area represents the region within a relative difference of 2% between the modeled dose and measured dose.

Figures 7 and 8 show the agreements and relative differences of the PDDs and the profiles for 3 × 3, 10 × 10, 30 × 30 cm^2^ fields between TPS calculations (CC and MC) and beam measurements for the 6XFF and 6XFFF modes. The shaded area represents the region within a relative difference of 2% between the modeled dose and measured dose. The PDD curves are well matched within 2% including the buildup region. Discrepancies of the profiles are generally within 2% in the high‐dose region and the low‐dose tail. The penumbra region shows a relatively greater deviation especially for larger fields but agrees with a distance to agreement (DTA) of no more than 1 mm for all fields. Both the CC and MC algorithms demonstrate good accuracies within the recommended tolerances of MPPG 5.a.,^23^ i.e., 2% for the high‐dose region, 3‐mm DTA for the penumbra region and 3% of maximum field dose for the low‐dose tail.

#### 4.B.2 MLC transmission factor and leaf‐tip offset

**Fig. 9:**
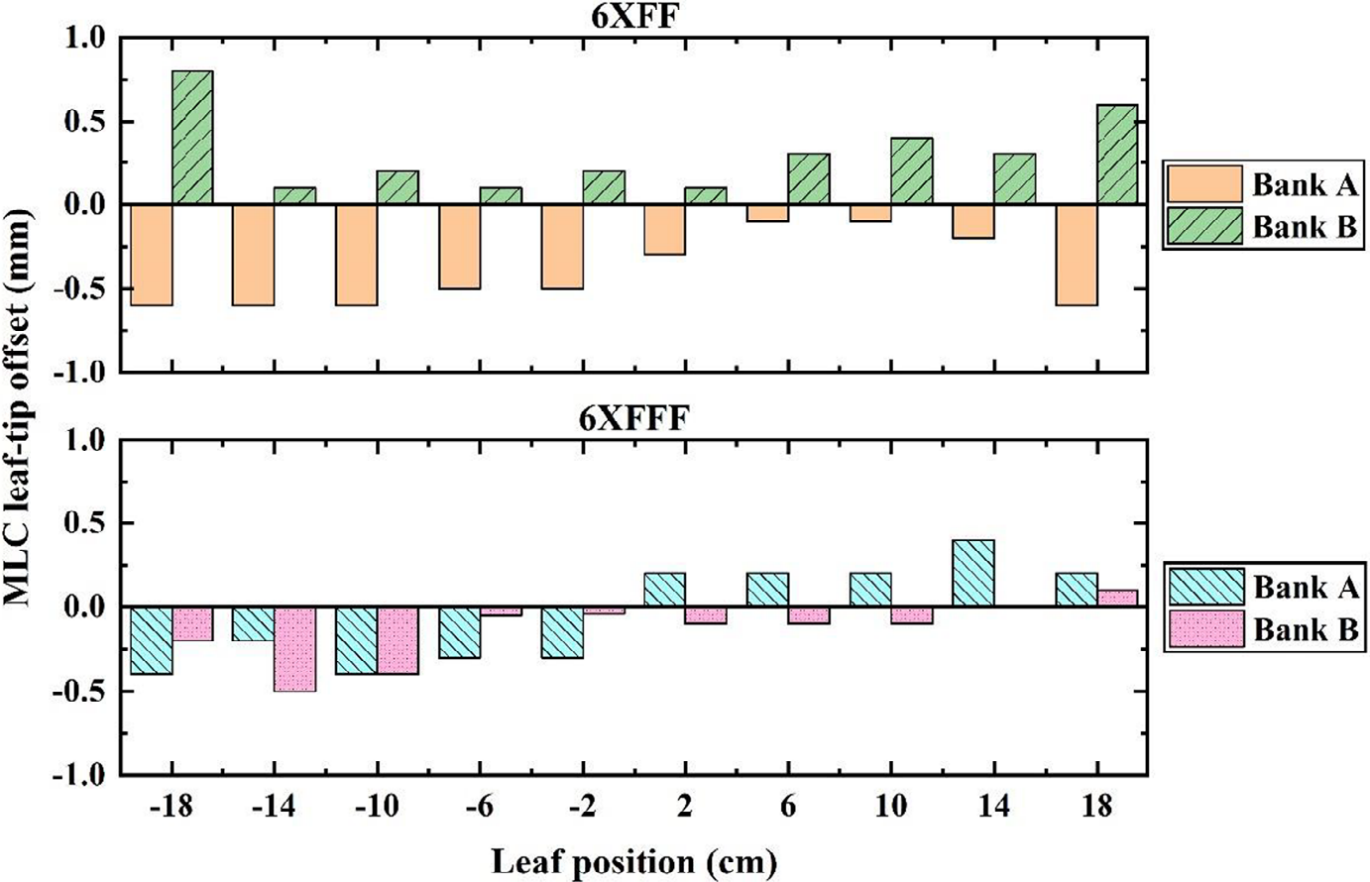
MLC leaf‐tip offsets of banks A and B at different leaf positions for 6XFF and 6XFFF.

The average MLC transmission factor was measured as approximately 0.9% for the 6XFF and 6XFFF modes, which indicated less than 2% tolerance as required in TG‐50.^14^ The measured MLC leaf‐tip offsets of the left and right banks at different leaf positions are shown in Figure 9. The offset for position between the measured points was determined by linear interpolation in beam modeling.

#### 4.B.3 Output factors

**Fig. 10:**
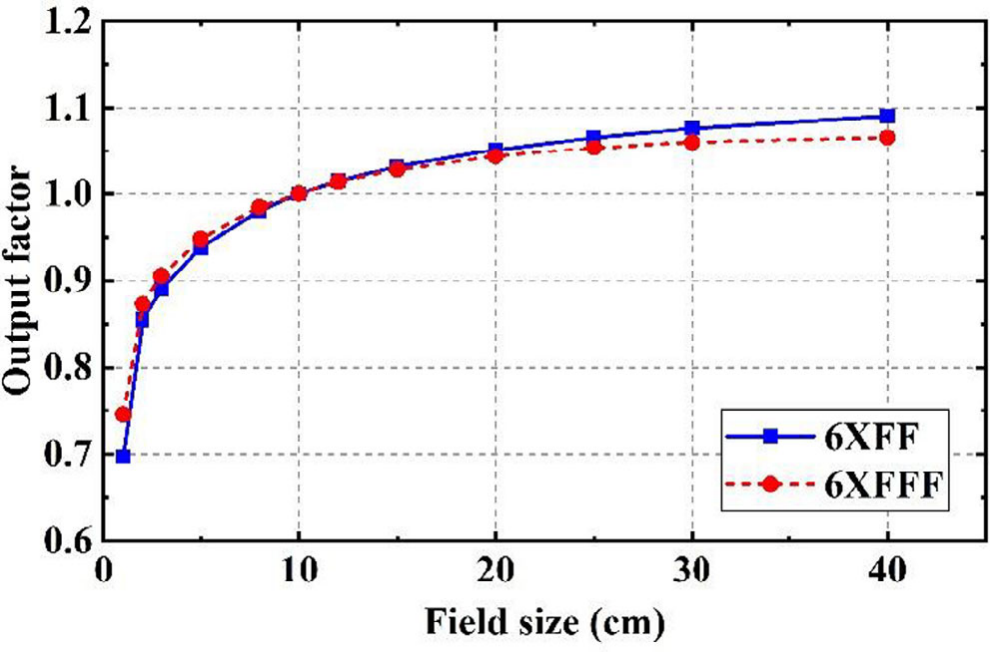
Output factors of 6XFF and 6XFFF beams.

Figure 10 shows the measured output factors of the 6XFF and 6XFFF beams. As mentioned earlier, the 5 × 5‐cm^2^ field was measured using both an ion chamber and a diamond detector and served as an intermediate transition for the output factor derivation of smaller field sizes using a “daisy chain” strategy.^34^

#### 4.B.4 Basic beam validation and IMRT commissioning

The basic beam validation involved photon tests for square, rectangular, asymmetrical, and irregularly shaped fields as well as for SSD dependence, virtual wedge and oblique incidence.^25^ In each test, a selection of central‐axis and off‐axis points were measured at various depths. Good agreements were observed between the measurements and corresponding calculated values within the recommended tolerances for different regions.^23^, ^25^

The TG‐119 tests used for IMRT commissioning included multitarget, prostate, H&N, and C‐shaped modalities.^19^ The commissioning evaluations were performed not only for sIMRT and dIMRT deliveries but also for uARC delivery in both 6XFF and 6XFFF modes. The TG‐119 defined DD was expressed as a ratio of prescription dose. For the CC method, the measured point DD ranged from −1.83% to 2.51% with an average of 0.59% for the high‐dose region and from −1.59% to 3.35% with an average of 0.59% for the low‐dose region. The MC method ranged from −1.15% to 3.14% with an average of 0.40% for the high‐dose region and from −1.74% to 2.84% with an average of 0.46% for the low‐dose region. For all the test cases, gamma passing rates of the two methods were better than 98% with criteria of 3% DD, 3‐mm DTA and 10% threshold, and around 95% with criteria of 2%/2 mm/10%.

### 4.C Imaging tests

#### 4.C.1 Image quality

**Fig. 11:**
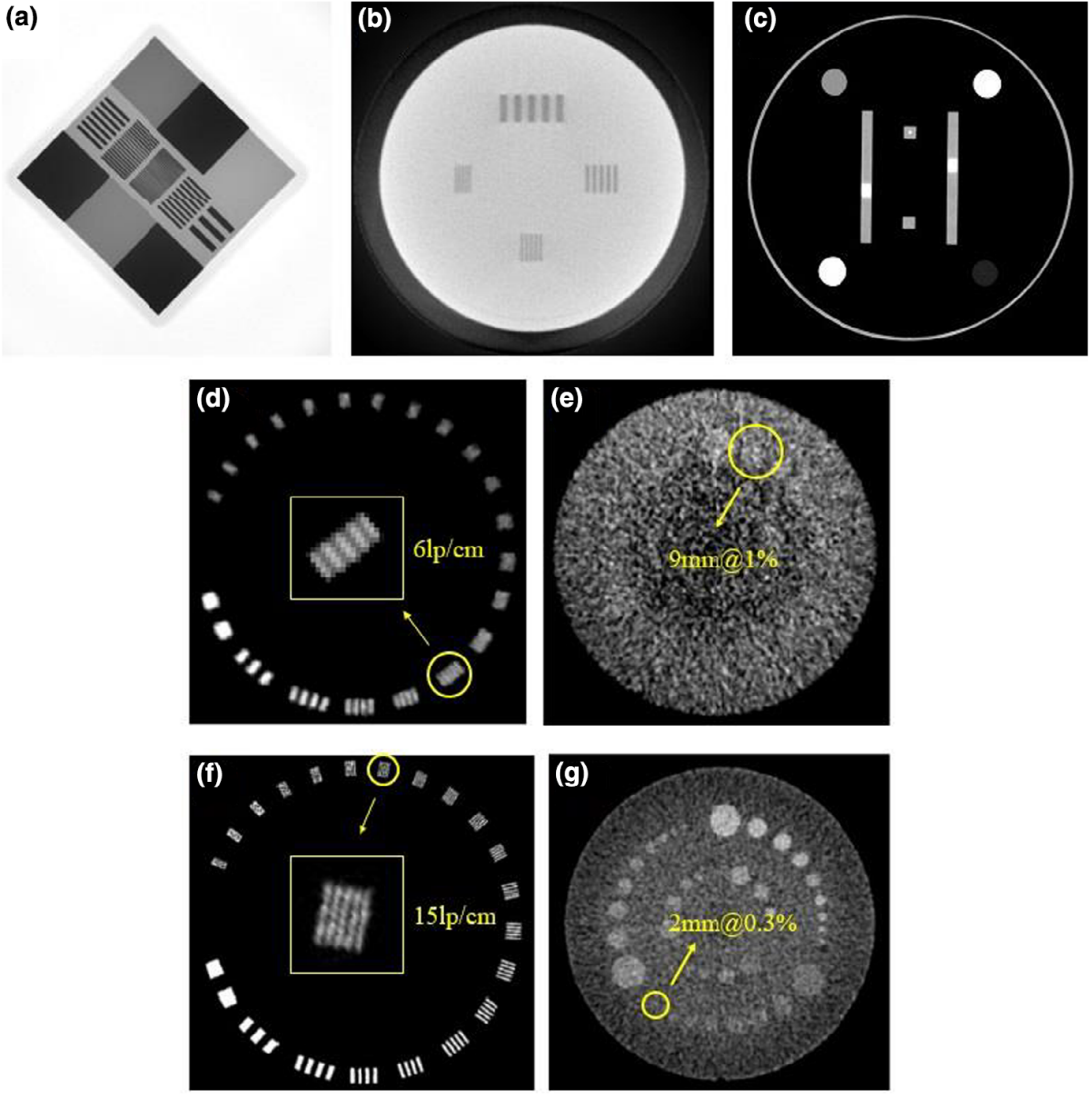
(a) Portal image of the 2DQA phantom, (b) CBCT slice of the 3DQA phantom, (c) FBCT slice of the CT system phantom, (d, e) CBCT slices and (f, g) FBCT slices of the CatPhan604 phantom.

Figure 11 shows the images of the 2DQA, 3DQA, CT system phantoms and the CatPhan604 phantom. The measured frequency of the 10% modulation transfer function (MTF f10) was 11 lp/cm for the portal imaging, 6 lp/cm for CBCT and ≥15 lp/cm for diagnostic FBCT (@120 kV, 120 mAs). The low contrast resolution was 9 mm for CBCT (1% @ 16 mGy) and 2 mm for FBCT (0.3% @ 40 mGy). For low‐dose protocols of FBCT, resolutions of 11 lp/cm and 8 mm (0.5% @ 3.5 mGy) were achieved with a reduction of dose by 90%. All the testing items were within the manufacturer's specifications.

#### 4.C.2 Geometric accuracy

The results of the WL test using the imaging beam were almost same with those with the treatment beam shown in Table 3 (within a difference of 0.1 mm), given the fact that the imaging and treatment beams were equivalently from the same source as explained Section 2. It suggested that the imaging isocenter of MV portal/CBCT, the treatment isocenter, as well as the mechanical isocenter agreed with each other within 1 mm. The result of panel alignment test using the orthogonal tungsten wires described in Section 3.D.2 showed that during gantry rotation, the positioning accuracy of EPID in the plane perpendicular to the beam axis was 0.30 and 0.03 mm in the crossline and inline directions, respectively, with a maximum rotation of 0.02° around the beam axis.

During the end‐to‐end IGRT accuracy testing of three modalities, the CBCT‐corrected setup errors were typically within 1 mm in three directions, which was a measure of the accuracy of geometric coincidence between the laser and CBCT imaging isocenter. On the premise of removing the setup errors, the couch corrections for three IG systems using the MIMI phantom with different loads and couch extensions are summarized in Table 4. The “isocenter” data are the “residual correction errors” after CBCT localization, indicating the automatic couch positioning accuracy (column of CBCT) and discrepancy between the linac and CT coordinates (column of FBCT). It is worth noting that at the CT position, couch top sag with full extension could be up to 15 mm, and couch flex in the lateral direction was up to 2 mm due to couch swing at 0° (less than 0.1°). By performing the real‐time correction, the overall IGRT accuracy was achieved at the submillimeter level under all conditions, mostly within 0.5 mm, in the superior–inferior (S‐I), left–right (L‐R), and anterior–posterior (A‐P) directions, which was sufficient to accommodate the needs of daily image guidance. The “predefined shift” data demonstrate the matching of the coordinate axes between each IGRT system and the reference CT.

**Table 4:** IGRT couch corrections using the MIMI phantom with different loads and couch extensions for the CBCT, FBCT, and MV portal systems.

Localization & Load	Couch extension	CBCT (mm)			FBCT (mm)			MV Portal (mm)		
		L‐R	S‐I	A‐P	L‐R	S‐I	A‐P	L‐R	S‐I	A‐P
Isocenter, 40 kg	Y ~ 15 cm	0	0	0	−0.4	0	−0.2	0.2	0.1	0
	Y ~ 55 cm	0.1	0	0.1	0.5	0.2	−0.3	0.3	0.1	−0.3
	Y ~ 80 cm	0	0	−0.1	0.1	−0.2	−0.2	0.3	−0.1	−0.2
Isocenter, 100 kg	Y ~ 15 cm	−0.1	0	0.1	−0.3	−0.5	−0.2	0.2	0.2	0.1
	Y ~ 55 cm	0.1	−0.1	−0.2	0.1	−0.1	−0.3	0.1	0	−0.3
	Y ~ 80 cm	−0.2	−0.2	0.2	0.2	−0.3	−0.2	0.2	−0.1	−0.2
Predefined shift (−12, −10, 14), 100 kg	Y ~ 80 cm	−11.8	−9.9	14.0	−11.5	−10.3	14.0	−11.7	−9.8	13.6

### 4.D End‐to‐end verification

The end‐to‐end tests were performed for breast (18 cases), H&N (nine cases), lung (six cases), rectum (three cases), cervix (three cases), and lung_SBRT (six cases) using sIMRT/dIMRT/uARC delivery techniques. The average point DD and gamma passing rate for each site‐and‐technique combination are listed in Table 5. The point DD of each plan was calculated as a ratio of the measured dose. The planar dose distributions obtained with the films were normalized to the maximum dose of calculation and analyzed using gamma criteria of 3% DD, 3‐mm DTA, and 10% threshold.

**Table 5:** Average point dose differences and gamma passing rates of collapse cone convolution (CC) and Monte Carlo (MC) methods for different sites and delivery techniques in the end‐to‐end tests.

Site	Delivery (case No.)	Point dose difference **(D_cal_. − D_mea_.)/D_mea_**.		Gamma passing rate **(3% DD/3‐mm DTA)**	
		CC	MC	CC	MC
Breast	sIMRT (6)	0.4%	1.6%	95.4%	93.2%
	dIMRT (6)	−0.2%	0.8%	94.3%	92.8%
	uARC (6)	−0.7%	0.2%	99.2%	97.2%
H&N	sIMRT (3)	1.4%	1.0%	97.7%	96.6%
	dIMRT (3)	1.9%	0.6%	96.2%	95.5%
	uARC (3)	1.3%	1.7%	96.7%	96.3%
Lung	sIMRT (2)	−0.2%	0.6%	96.4%	97.9%
	dIMRT (2)	−1.4%	−0.6%	96.5%	97.4%
	uARC (2)	−1.7%	−0.4%	96.3%	96.4%
Rectum	sIMRT (1)	−1.1%	−1.2%	95.2%	96.9%
	dIMRT (1)	−0.9%	−1.4%	96.4%	98.4%
	uARC (1)	−1.4%	−0.3%	92.6%	92.2%
Cervix	sIMRT (1)	−0.9%	−0.8%	99.72%	99.67%
	dIMRT (1)	0.1%	−0.3%	99.69%	99.64%
	uARC (1)	−1.7%	−1.4%	99.32%	99.13%
Lung_SBRT	sIMRT (2)	1.1%	1.4%	97.70%	96.60%
	dIMRT (2)	1.1%	1.7%	99.33%	98.52%
	uARC (2)	1.6%	1.9%	97.19%	97.53%

**Fig. 12:**
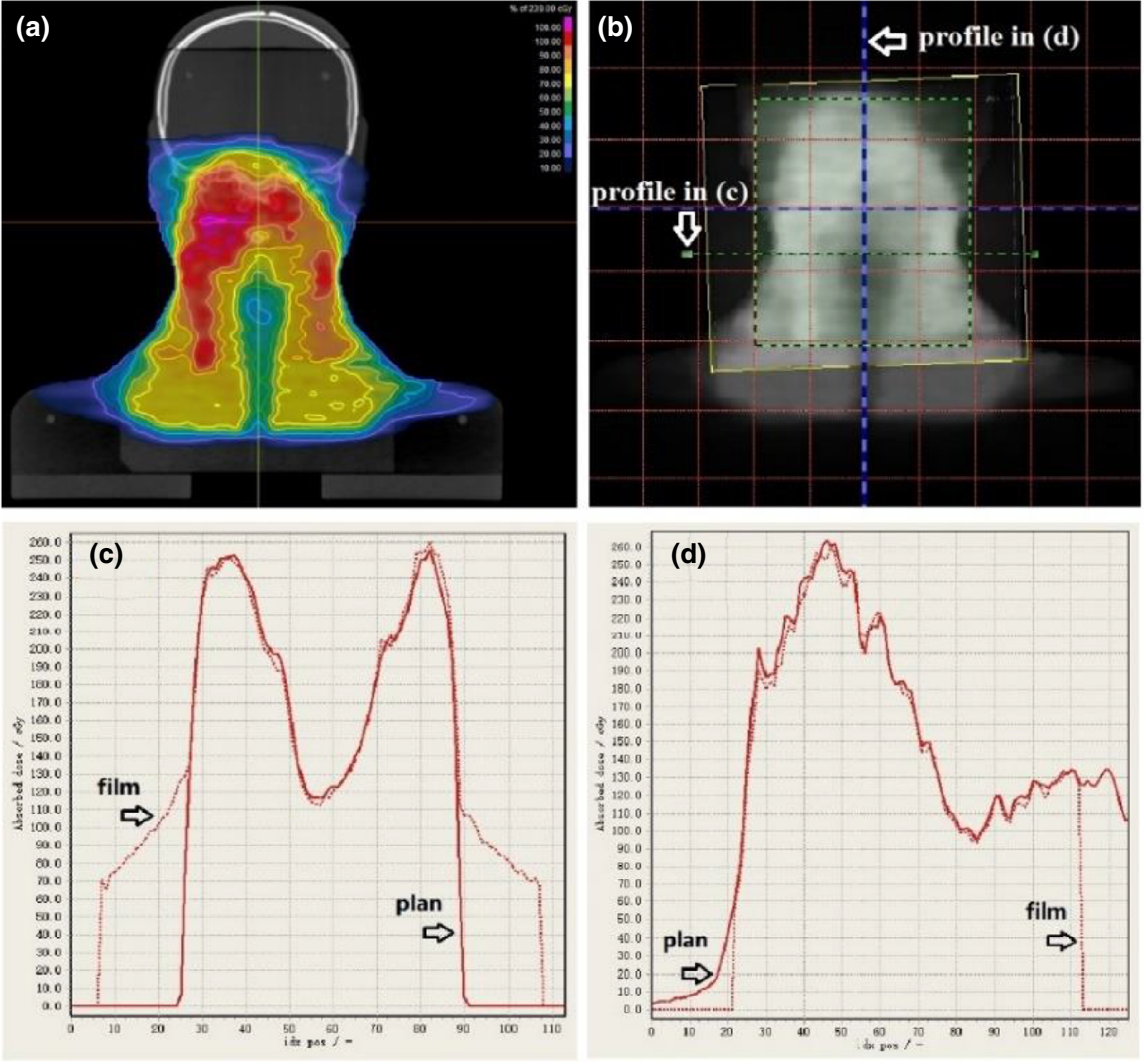
End‐to‐end test of an H&N case using the CIRS head and shoulder phantom. (a) Planned dose distribution (CC calculation) at the level of film on the coronal plane. (b) Dose map of the exposed film overlapped with the planned dose. (c) Corresponding horizontal and (d) vertical profiles of planned dose (solid) and measured dose (dotted).

Figure 12 shows an example of an end‐to‐end H&N case with nine‐field sIMRT beams. In this case, a gamma passing rate of 97.7% for the CC algorithm and 96.3% for the MC algorithm was achieved with 3%/3 mm/10%.

### 4.E Routine QA results

#### 4.E.1 Temporal stabilities of machine output and IGRT accuracy

**Fig. 13:**
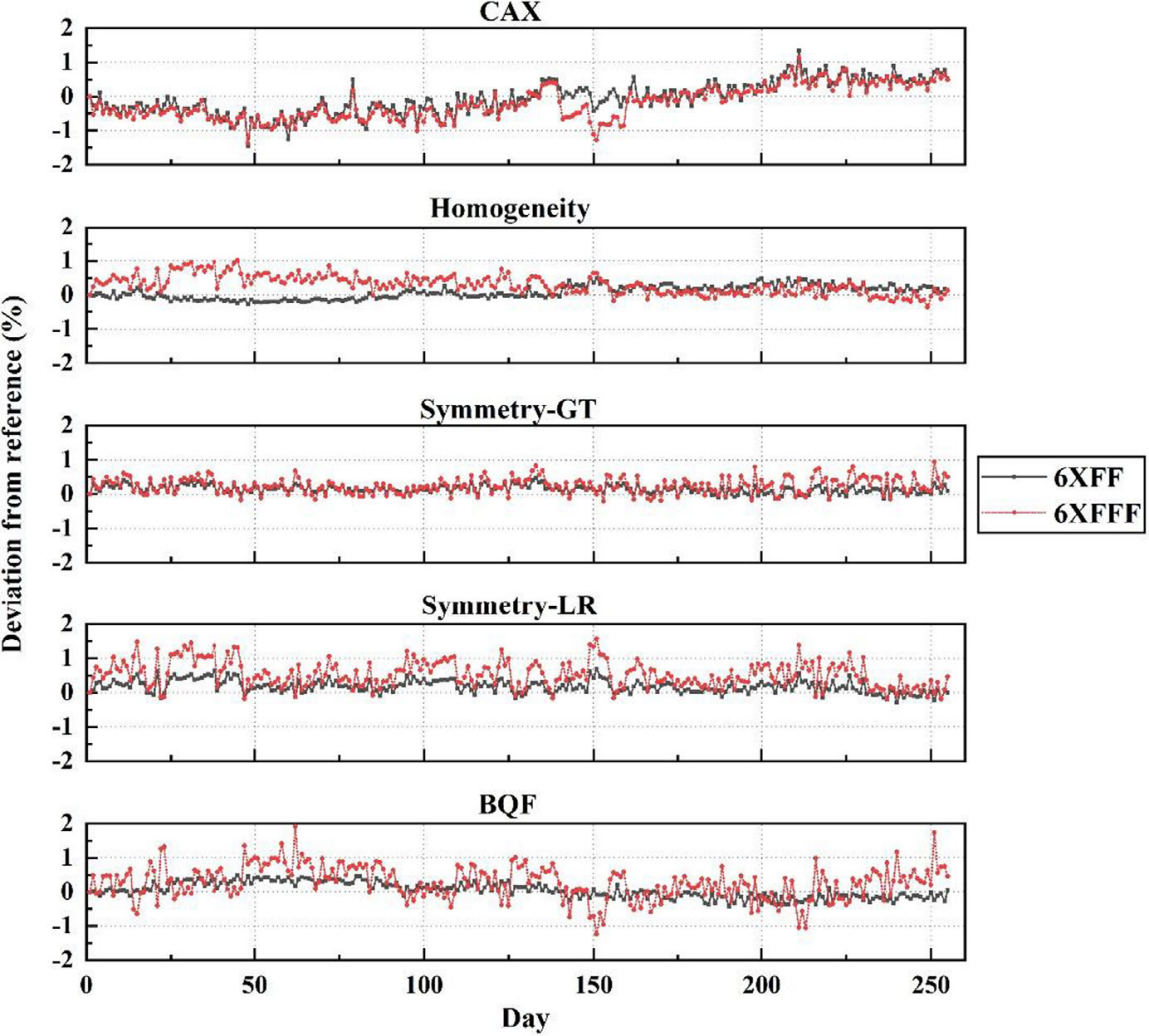
Daily output check of uRT‐linac 506c over 1‐year period.

Since the uRT‐linac 506c platform was commissioned and prepared for clinical operation in November 2019, the stabilities of daily output, homogeneity, symmetry, and beam quality factor (BQF) have been monitored using the PTW QuickCheck device, as shown in Figure 13. Over a period of 1 year, the deviation of the central axis output (CAX) from the reference was −0.07 ± 0.48% for 6XFF and −0.21 ± 0.47% for 6XFFF, with a slight positive drift of approximately 0.1%/month. Machine output was retuned on around day 140 after a major repair. Variations in homogeneity, symmetry and BQF were found to be less than 1% except for the symmetry‐LR and BQF of 6XFFF. The larger discrepancies (still within 2%) of symmetry and BQF measurements for 6XFFF might result from their sensitivity to detector positioning error due to the higher gradient dose profile of FFF beams.

**Fig. 14:**
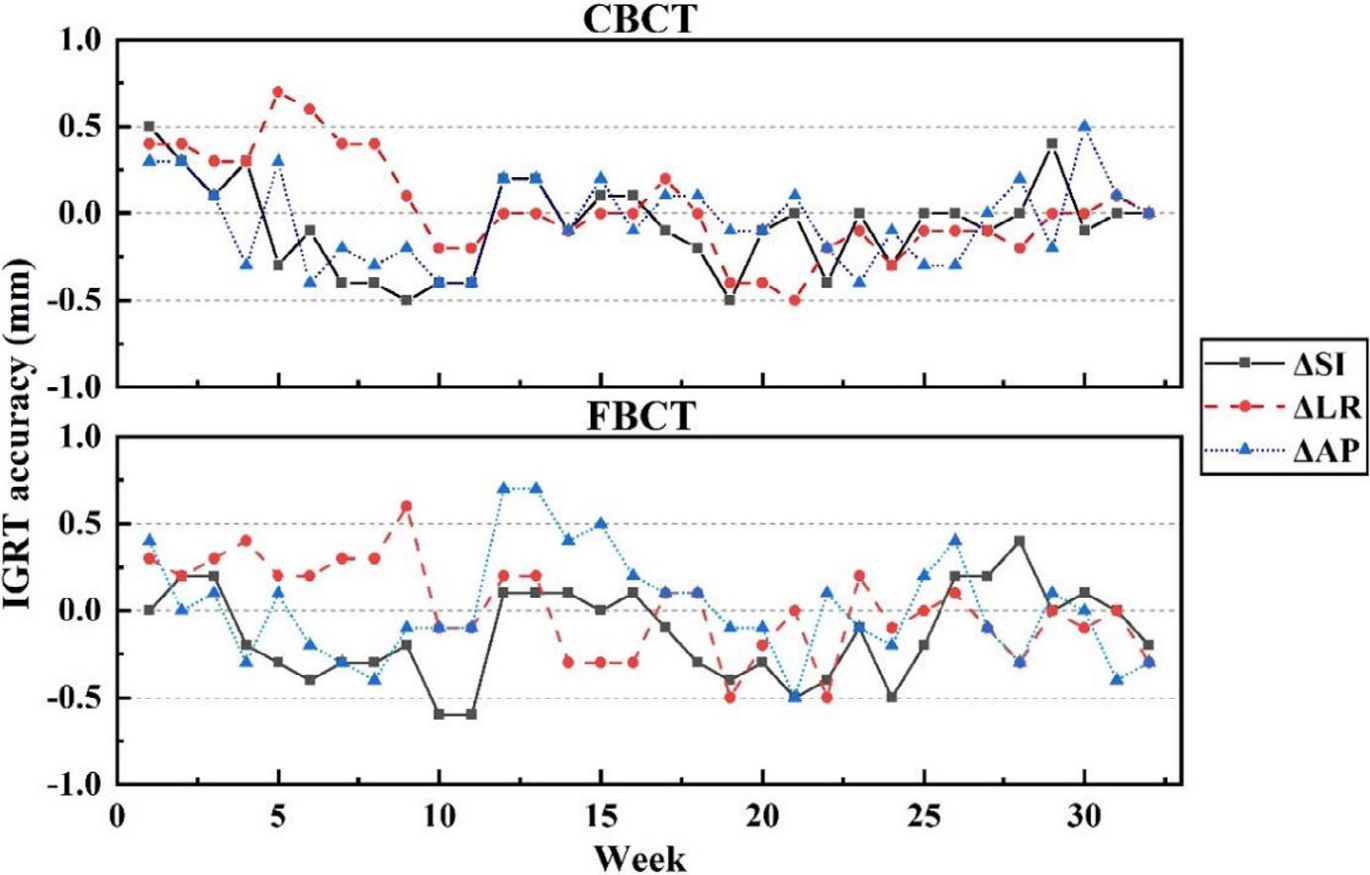
Weekly QA results of IGRT accuracy with CBCT/FBCT on uRT‐linac 506c.

The IGRT accuracies of volumetric imaging modalities were checked in a routine weekly QA, following the commissioning procedures, i.e., Steps (1)–(3) in Section 3.D.2, but without putting weight load. The weekly QA results of IGRT accuracy for CBCT and FBCT are plotted in Figure 14. It shows that the CT‐IGRT accuracy demonstrates good stability and reproducibility within the vendor’s specification of $\sqrt{{\left(\Delta \mathrm{SI}\right)}^{2}+{\left(\Delta \mathrm{LR}\right)}^{2}+{\left(\Delta \mathrm{AP}\right)}^{2}}\leq 1\;\mathrm{mm}$.

#### 4.E.2 Patient‐specific QA

At the time of submission, over 600 patients were treated for sIMRT on the uRT‐linac 506c, and nearly 100 patients were treated for dIMRT and uARC. The point DD was within 2% for sIMRT plans and within 3% for dIMRT/uARC plans. Gamma passing rates of 3D dose distributions were better than 99%/plan and 95%/beam (3% DD/2 mm DTA/10% threshold) for all plans.

## 5 DISCUSSION

### 5.A Initial clinical experience in use of the uRT‐linac 506c platform

Since the new platform started its first clinical treatment in January 2020, the uRT‐linac 506c demonstrates good performance in dosimetric accuracy and treatment efficiency. Benefits from two clinical innovations deserved to be mentioned. The first is the capability of 1.5 revolutions of linac gantry. Removing the limit at 180° means no need of idle rotation, which is more efficient in circumstances like treating a posterior target with uARC technique, or being able to take a full‐circle scan of CBCT starting from any angle. The second is the integrated CT system. Seventy centimeters in diameter of the CT bore is generally adequate except for breast and lung patients with arms up in large degrees. Compared with the cone‐beam geometry, the image quality of FBCT is less affected by scatter radiation in patient that reaches the detector, thus has a better CNR and low‐contrast detectability. The advantage of IGRT employing the kV‐FBCT lies in the improvement of target localization accuracy during soft tissue registration, which has been discussed in previous studies. Morrow et al.^35^ reported that superior image quality with the kV‐FBCT resulted in reduced uncertainty in soft tissue registration during IGRT compared with other image modalities, especially for prostate and gynecological tumors surrounded by soft tissues. Peng et al.^36^ quantitatively characterized the interfractional variations of organs for prostate cancer, and their dosimetric effects were directly evaluated based on daily kV‐FBCT data. In our clinical practice, compared with the kV‐CBCT, the kV‐FBCT provides a better visualization of soft tissue structures with faster scanning speed, fewer motion artifacts, more accurate CT number, and longer scanning range and may benefit the patients with thoracic and abdominal tumors. In addition, we found some non‐rigid changes of patients' anatomy in either morphology or physiology, which could not be corrected by a translational couch shift. The need for adaptive replanning should be evaluated in such circumstances. Online ART is expected in the near future by employing auto‐segmentation and auto‐planning based on daily FBCT.

### 5.B uRT‐linac 506c vs. CT‐on‐rails

The integration of a diagnostic‐quality CT with a linac is not an original design. The CT‐on‐rails system has set a precedent for in‐room CT imaging; however, it is not commercially available any more, mostly because of its doubtful accuracy and complex workflow for IGRT. Extra uncertainties may be introduced during couch rotation and CT gantry movement. Although the overall mechanical precision is predicted to be within 1 mm by evaluating all the sources of potential uncertainties,^9^ as far as we know, vibration or miscalibration of the CT gantry moving on rails could lead to either poor image quality or spatially displaced objects, and the accuracy of patient alignment may be subject to variations of mechanical flex, couch sag, and positioning accuracy during couch rotation. That is why a fiducial marker method is recommended in the alignment workflow in order to transfer the isocenter information from the linac side to the CT images. By contrast, the uRT‐linac 506c system exhibits new features in geometry and clinical workflow. It suggests a unique configuration in which the CT scanner is fixed behind the linac gantry. The long couch traveling distance to CT position has little impact on the geometric accuracy between the linac and CT coordinate coincidence, as we have validated earlier, which is attributed to the real‐time correction of CT coordinates. It is of great importance to assure the reproducibility of geometric accuracy. And the CT‐IGRT procedure on the uRT‐linac 506c seems more efficient, as described in Figures 3 and 4. Generally, it takes approximately 1 min for the whole process, which is comparable to the standard kV‐CBCT procedure.

## 6 CONCLUSION

This study summarized the commissioning process of a new fully integrated CT‐linac, the uRT‐linac 506c, and preliminary experiences in clinical operation. The commissioning and QA results indicate that this treatment platform exhibits good performance in dosimetric and mechanical accuracies. As the first clinical model type, its long‐term reproducibility and stability are still under inspection. The integrated CT system, as a highlight, allows a diagnostic‐quality visualization of internal patient anatomical structures for accurate image guidance with a concise workflow, and paves the way towards online ART.

## CONFLICT OF INTEREST

The authors have no conflict of interest to disclose.

## AUTHOR CONTRIBUTIONS

Lei Yu and Jun Zhao performed the measurements and wrote the manuscript with the aid of Jiazhou Wang in interpreting the results. Zhen Zhang and Weigang Hu supervised the project and were in charge of overall direction and planning. All the authors provided critical feedback and contributed to the final version of the manuscript.

## Data Availability

The data that support the findings of this study are available from the corresponding author upon reasonable request.
